# Vestibular and Multi-Sensory Influences Upon Self-Motion Perception and the Consequences for Human Behavior

**DOI:** 10.3389/fneur.2019.00063

**Published:** 2019-03-07

**Authors:** Zelie Britton, Qadeer Arshad

**Affiliations:** Department of Neuro-Otology, Charing Cross Hospital, Imperial College London, London, United Kingdom

**Keywords:** self-motion perception, vestibular system, cerebellum, cortex, behavior

## Abstract

In this manuscript, we comprehensively review both the human and animal literature regarding vestibular and multi-sensory contributions to self-motion perception. This covers the anatomical basis and how and where the signals are processed at all levels from the peripheral vestibular system to the brainstem and cerebellum and finally to the cortex. Further, we consider how and where these vestibular signals are integrated with other sensory cues to facilitate self-motion perception. We conclude by demonstrating the wide-ranging influences of the vestibular system and self-motion perception upon behavior, namely eye movement, postural control, and spatial awareness as well as new discoveries that such perception can impact upon numerical cognition, human affect, and bodily self-consciousness.

## Introduction

Despite the vestibular system being evolutionarily ancient (1), it has long been overlooked as a primary sensory organ, notably by Flourens who, whilst identifying that pigeons with peripheral vestibular lesions suffered from imbalance, concluded that the semi-circular canals were involved in generating motor responses for head and eye movements (2). The inner ear itself was first recorded in the 1,500 s by Andreas Vesalius and Gabriele Fallopio, reviewed by Weist (3). Initial research into the mechanics behind how acceleration can be detected took place in the 1870s by three independent scientists: Josef Breuer, a Viennese doctor, Ernst Mach, a professor of physics and Alexander Crum Brown, who worked as a chemist having received degrees in medicine and chemistry. They identified the semi-circular canals as the organs for motion sensation, suggested relative inertial motion of endolymph to the bony skull as the method of transduction, and observed that the semi-circular canals and otoliths might work in combination to differentiate between linear motion and tilt, and whose work forms the basis of our current understanding, well-reviewed by Weist and Baloh (4) and Weist (3). The vestibular system is found in different forms across the animal kingdom and is reviewed by Lowenstein (5) and Beisel et al. (6).

The paired vestibular organs consist of three semi-circular canals and two otoliths, which together sense rotational and linear accelerations and are responsible for maintaining both stable vision during head movements [via the vestibular-ocular reflex (VOR)] and a stable posture (via vestibular-spinal reflexes). Furthermore, they also contribute to an awareness of our movement in space as demonstrated by the ability of a subject to report passively applied movements whilst seated in a rotating chair in darkness. In everyday life, vestibular stimuli are integrated with visual, somatosensory, auditory, and motor efference inputs to derive estimates of self-motion. Perhaps the reason for the omission of vestibular perception from the traditional human senses is that, compared with the perceptual times for other senses, vestibular perceptual awareness is relatively slow (70–160 ms) and less sensitive (7, 8). Accordingly, during daily life we are often unaware of workings of the vestibular system until it fails. Patients with vestibular disorders suffer not only from difficulties with balance but also report head-movement induced oscillopsia and difficulties during complex behaviors such as self-motion perception and navigation (9). This review will examine the role of the vestibular system in the perception of self-motion and explore how self-motion perception can modulate other behaviors.

## Sensing Motion

The vestibular organs are our motion detectors and consist of the otoliths and the semi-circular canals. These detect changes in velocity via stimulation of the hair cells which contain cilia projections from their apical surface. The cilia are named according to their length: the longest being the kinocilium, the others, the stereocilia. Even in the absence of any stimulation, they exhibit a low level of tonic activity (10–12). Hair cells depolarise when the stereocilia deflect toward the kinocilium and hyperpolarise when the deflection of the stereocilia is directed away from the kinocilium (13–15). Depolarisation leads to release of neurotransmitters onto first-order vestibular neurons. Such deflections occur due to the relative inertia of the endolymph in the semi-circular canals into which the cilia project: when the head accelerates, the lag of this fluid deflects the cilia. The hair cells also receive efferent synapses which can modulate the activity of the hair cells (16). The utricle and saccule are the two otoliths and detect linear accelerations in the axial and coronal planes, respectively. Their hair cells project into a gelatinous layer which is covered with calcium carbonate crystals. The anterior, posterior, and horizontal canals work in pairs to sense rotations in the sagittal (pitch), coronal (roll), and transverse (yaw) planes, with an increase in impulse discharge during ipsilateral rotation and a reduction seen during contralateral rotation (11) [it might be added here that the semi-circular canals have also been shown to respond to tilting and linear acceleration, albeit with a much greater threshold (17)]. Two distinct types of afferent neurons, categorized by the regularity of their resting activity spike pattern, carry signals from the hair cells to the vestibular nuclei (12). For canal afferents, regular fibers, which have smaller axon diameters, are thought to predominantly transmit information about head motion over time whereas the irregular fibers are more sensitive to motion, exhibiting higher gain (18, 19). Both fibers respond similarly to active and passive head motion (20). Otolith afferents are similarly formed of regularly- and irregularly-firing neurons (21).

## The vestibulo-ocular Reflex

The vestibulo-ocular reflex (VOR) serves to stabilize visual input on the retina during short, fast head movements by driving the eyes with a velocity of equal magnitude and in opposite direction to the head movement. It was first described by Andreas Hoegyes who demonstrated that each semi-circular canal was connected to the appropriate extra-ocular muscle (22). The canal afferents, having tonic discharge that is modulated according to the direction of rotation (10, 11), work in pairs, such that stimulation of one side occurs whilst the other side is inhibited (23). Similar mechanisms exist for translational head movements, which result in a linear VOR (24, 25). The VOR is fittingly fast, operating with latencies of 5–6 ms (26), which is in keeping with the short three-neuron pathway involved: the primary afferent neuron of the vestibular nerve, an interneuron, and a motor neuron to the corresponding extra-ocular muscle (27–29). The functional importance of which was first recognized by Lorente de No, who discovered that feedback pathways within the neuronal arc are involved in the VOR, a concept extended by Raphan et al. in their description of the velocity storage mechanism (30, 31). Furthermore, the VOR is sensitive, and can respond, to changes in the relationship between vestibular signals and the visual field: wearing magnifying lenses leads to adaptive increases in VOR gain whilst left-right reversing Dove prisms lead to adaptive decreases in the gain of the VOR (32–34). The mechanism for these adaptations appears to be via long-term depression in the cerebellar flocculus (35, 36). [Note that removal of the vestibulocerebellum does not abolish the VOR (34)]. Following unilateral vestibular loss, there is an impressive recovery of the VOR, revealing the importance of multimodal input integration, in particular, proprioceptive, and motor efferent inputs (37, 38). Nystagmus arises when there is slow, continuous movement of the head, with the slow, vestibular, component in the opposite direction of the motion, and a fast, “catch-up” saccade in the same direction [note that nystagmus can, of course, arise in other circumstances, namely: physiological nystagmus (optokinetic and end-point); infantile nystagmus and pathologic nystagmus, reviewed by Abadi (39)]. Early work carried out by Lorente de No established the importance of the role of the reticular nuclei in these reflexes: in rabbits with lesions of the raphe nuclei of the pons and the medulla oblongata, thus severing the axons of the reticular nuclei, the fast component of nystagmus disappeared (40, 41).

## What Is Self-Motion Perception and How Does the Vestibular System and Associated Central Processing Give Rise to it?

### Perception of Angular Motion

**Figure 1:**
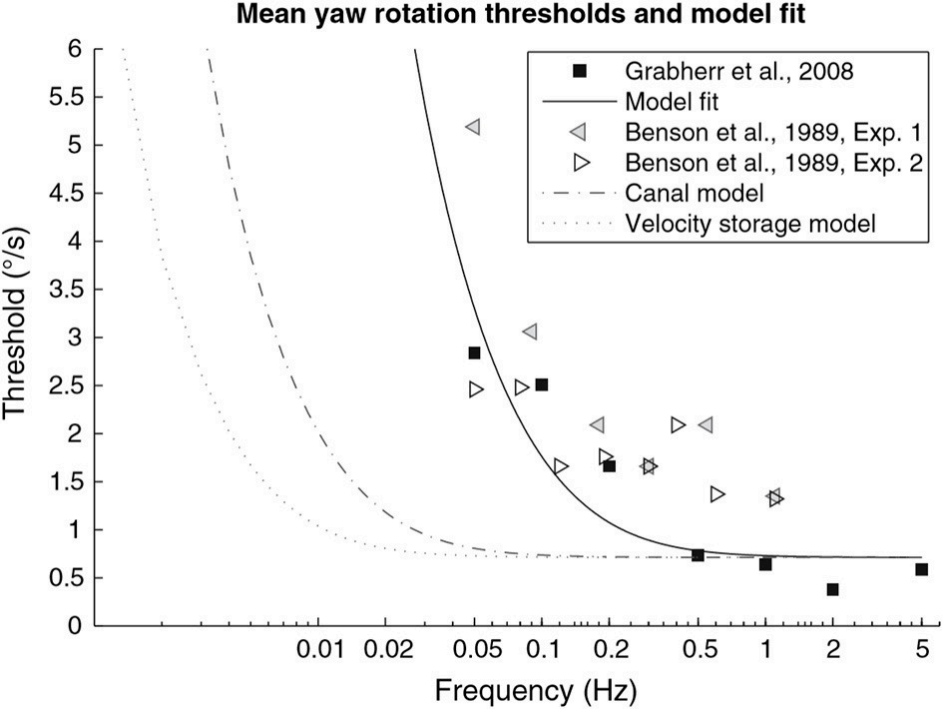
From section Perception of angular motion. This figure, modified from Grabherr et al. (43). Graph showing velocity thresholds as a function of sinusoidal motion frequency, where velocity is the peak velocity achieved in each cycle of sinusoidal acceleration. Black squares represent mean data from Grabherr et al. (43), *n* = 7. Left and right pointing triangles from Benson et al. (42), *n* = 6 and *n* = 8, respectively. Solid black line represents the fitted model for the high-pass filter K_TS_/(TS + 1).

Perception of passive self-rotation can be quantified in terms of the minimum (or threshold) rotation required for perceptual awareness and by a subject's estimates of angular velocity and/or displacement. Vestibular perceptual thresholds are dependent upon the axis of rotation, with thresholds for whole-body rotations about the vertical axis (yaw) being significantly lower than those for roll and pitch (42). Additionally, perceptual thresholds improve as the frequency of sinusoidal rotation increases up to 0.2 Hz, and plateau beyond 0.5 Hz, findings that suggest that vestibular signals undergo high-pass filtering (see Figure 1) (43). Vestibular perception thresholds for yaw rotations in young healthy subjects are significantly greater at 1.18 deg/s^2^ compared with the angular acceleration required for nystagmus (0.51 deg/s^2^) (44).

Eye movements can also be used to indicate perceived rotation by implementing a paradigm in which participants are either asked to hold a given direction of gaze in the dark during angular rotation (which requires both the vestibular-ocular reflex and a compensatory saccade), or are instructed to make a saccade back to a previously seen visual target after having been rotated in the dark (vestibular memory-contingent saccade) (45). Subjects perform marginally better in the latter, possibly secondary to interference between the VOR and the rotation being estimated during the former task (46). As with threshold perceptions, yaw rotations yield the best accuracy (46). Labyrinthine-defective patients are unable to produce any structured response as would be expected of a task designed to test vestibular perception (47).

The ability to estimate and reproduce rotational *displacement* is another method to probe vestibular perception. Metcalfe and Bronstein examined the ability of patients with labyrinthine disease and healthy controls to re-orientate themselves using a self-controlled motorized Barany chair after passive displacement in the dark in a “go-back-to-start” paradigm (48). Controls demonstrated high accuracy with low degrees of variation (5–15° for 30–180° displacements). Patients with acute unilateral vestibular failure (within a month of symptom onset) demonstrated an inability to accurately perceive rotations in either direction, consistently underestimating magnitude of displacement toward the lesion and exhibiting highly variable responses to rotation in the opposite direction. The study followed the patients up for several months, by which time there was partial restoration of perception and the symmetry of the responses had been restored, suggesting compensatory central mechanisms.

More recently, Panichi et al. sat subjects in a head-fixed rotating chair in darkness and asked them to fixate on the location of a previously seen target (presented straight ahead prior to rotation) (49). The chair rotated in an asymmetric sinusoidal pattern, with a fast component in one direction and a slow, restoring component in the opposite direction, an arrangement previously shown to selectively bias central vestibular perceptual processing (50). They found that patients with acute vestibular neuritis have a large deficit in vestibular perception during conditions in which the slow-phase acceleration was toward the lesioned side and that whilst this improved over the 1-year follow-up period, it did not return to normal. Notably, this asymmetry of self-motion perception correlated with patients' dizziness handicap inventory score. It has been well-documented that clinical outcomes in patients with chronic dizziness correlate poorly with low-level brainstem reflexes (i.e., VOR) (51–53) and much better with cortical processes including visual dependence and anxiety and depression (54, 55). These observations provide support for the theory that there exist different central mechanisms for compensation of VOR and vestibulo-perceptual responses, with the latter higher-level processes affording better predication of prognosis following vestibular dysfunction.

### Perception of Linear Motion

**Figure 2:**
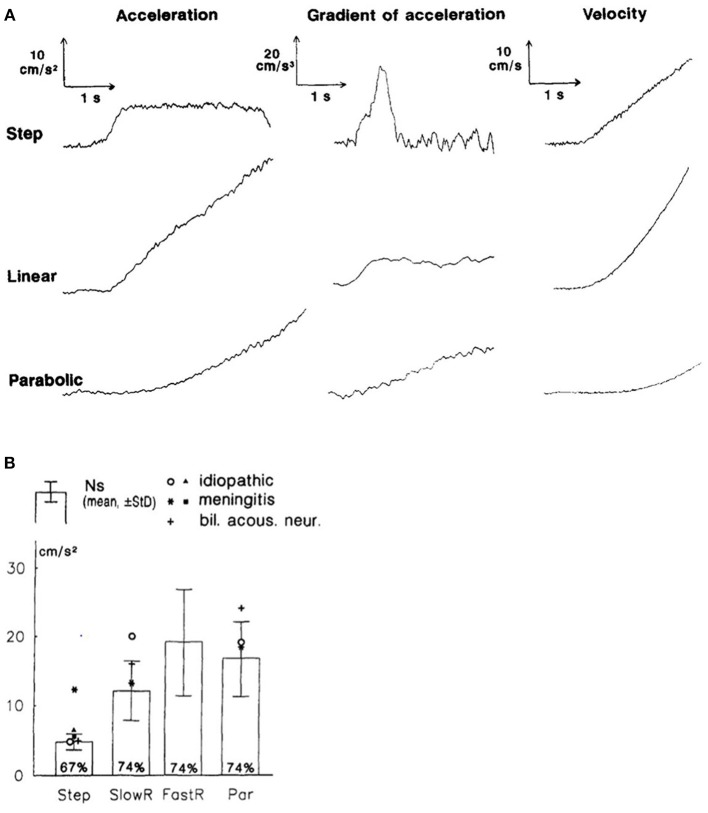
From section Perception of linear motion. This figure, modified from Gianna et al. (60). **(A)** Motion profiles for acceleration steps and corresponding rate of change of acceleration and velocity. **(B)** Acceleration thresholds for normal subjects (Ns) (mean +/– standard deviation) and individual subjects with vestibular impairment in the different conditions: step accelerations, low linear ramp (SlowR), high linear ramp (FastR), and parabolic acceleration (Par).

Linear accelerations are sensed by the otolith organs, and the double integral of their signal can be used to estimate passive linear displacement in the absence of other sensory inputs (56, 57). In their study, Israel et al. found that whilst subjects were unable to spontaneously produce a passive linear displacement of two meters when blindfolded, they were able to reproduce the distance traveled, peak velocity, and velocity profile following passive displacement and that, in this paradigm, reproduction of parameters relating to velocity appeared to have been processed independently of the reproduction of displacement (56). Regarding vestibular perception of linear motion, lateral movements have lower thresholds than anterior-posterior movements: in one study using a sinusoidal stimulus of frequency 1 Hz, thresholds for accelerations were 6.5 cm/s^2^ and 8.5 cm/^2^ for lateral and anterior-posterior movements, respectively, whilst thresholds for velocity were 10.4 and 13.5 cm/s (58). Vertical linear movements have a perceptual threshold greater than that for lateral movements but less than that for anterior posterior motion (59). Using single acceleration steps, Gianna et al. found acceleration thresholds of 4.84 cm/s^s^ and velocity thresholds of 7.93 cm/s for lateral movements (60). Other movement profiles with linear and parabolic ramping of the acceleration resulted in higher thresholds, thereby supporting the view that large acceleration gradients facilitate perception (see Figure 2). The study also examined the thresholds of patients with impaired vestibular function. Although their average thresholds were worse than healthy controls, there was overlap between the two groups, suggesting that somatosensory cues were also used in the task. A recent paper examined the effects of a central (vestibular migraine) and peripheral (Menière's disease) vestibular dysfunction on linear motion perception, finding that perceptual thresholds were higher for patients with Menière's disease but not significantly different for vestibular migraine patients compared with controls (59). These findings contradict recent findings of abnormal tilt thresholds in vestibular migraine patients (61).

### Perception of Heading

The ability to estimate one's direction of translation is termed heading perception and the vestibular system plays an important role in this process. For example, in macaques heading discrimination thresholds in the dark increase 10-fold after bilateral labyrinthectomy (62). Regarding the relative contribution of the visual and vestibular systems in heading estimation tasks, it appears that when subjects are asked to point out the heading direction, the visual system enables more precise determination (63), but when asked to perform a discrimination task (forced choice of two), thresholds are similar for the two senses (62). Interestingly, body position relative to gravity can modify vestibular heading perception, but visual heading perception is unaffected by changes in body position (64).

### Calculating Self-Motion Relative to the World

Signals generated by the vestibular system create an egocentric reference of self-motion: to be useful for guiding our movements and behavior relative to the external world, a transformation to an earth-referenced frame of self-motion is required. To create an earth-referenced model of self-motion, two difficulties need to be overcome. Firstly, the signal from the semi-circular canals does not vary with the attitude of the head in space, for example, a raw rotation generates the same signals at the level of the hair cells whether the subject is upright or supine. Secondly, the otoliths alone cannot distinguish linear acceleration from head tilt relative to gravity (65). These difficulties can be overcome through integration of vestibular signals with additional inputs including visual and proprioceptive stimuli (reviewed below). However, even when undergoing passive motion in a dark room, a solution can theoretically be computed by combining information from the canals and the otoliths. To resolve these problems, it has long been hypothesized that the brain calculates an estimate of the attitude of the head relative to gravity using multisensory inputs, including canal signals, a value that can then be used to resolve the above issues (66). In monkeys undergoing passive movements, some cerebellar nodulus/uvula Purkinjie cells respond preferentially to translation (or rather, to the vector perpendicular to gravity) (67) whereas others respond to tilt (68). These neurons project to the vestibular nuclei and the fastigial nucleus, and from there to the thalamus, which also demonstrates varying degrees of separation of movement types relative to gravity (69, 70). Modeling work suggests a similar mechanism exists in humans (71).

### Differentiating Between Actively-Generated and Passively-Applied Motion

An unaddressed question is how we differentiate active vs. passive motion. In life, we experience a combination of actively-generated and passively-applied motion. Yet the relative movement of the endolymph, and the subsequent deflection of the hair cells, is identical during both active and passive movements of a given profile and acceleration. The question arises as to how changes in sensory signals due to external variables (exafference) vs. those resulting from our own actions (reafference) can be distinguished. An in-built mechanism would be to use a copy of any motor commands against which to compare sensory stimuli: this exists in the form of discharge corollaries, also known as motor efference copies. Subtraction of the actual sensory signal from the predicted sensory result of an action theoretically leaves the signal from any additional passive motion.

As noted previously, in alert primates, semi-circular canal afferents respond identically to actively- and passively-generated head movements (20). In contrast, vestibular nuclei neurons show differential activation to passive and active head movements, with reduced responsiveness to vestibular afferents during actively-generated movements (72–74). In an experiment designed to probe the mechanism for such modulation of vestibular neurons responsiveness, Roy et al. compared the activity of medial vestibular nuclei neurons during a range of tasks including: passive whole-body rotations; active head movements; passive body rotations, controlled by the monkey using a steering wheel to drive a turntable, with an earth-fixed head (to activate neck proprioceptors); and, head restrained monkeys actively trying to turn their heads (motor commands but no corresponding proprioceptive signals) (75). Only during the actively generated head movements did the authors observe a reduction in vestibular nuclei neuron responsiveness to vestibular afferent signals. Furthermore, in the paradigm where the monkeys were attempting but unable to move their heads (i.e., there was muscular activation but not a corresponding change in muscle length and joint movement), there was minimal modulation of vestibular nuclei neuron responsiveness. Taken together, these observations suggest that motor efference copies and not proprioceptive signals nor prior knowledge of the movement that lead to suppression of vestibular neuron responses during actively generated movements, and that only when the motor efference copy matches the proprioceptive input does reafference occur. As a neural correlate of this, during active self-motion, neurons in the fastigial nucleus continually compare predicted and actual sensory stimuli (76) and respond only to unexpected self-motion (77). Neurons in the posterior parietal cortex also exhibit a differential response to active and passive movements, although the responses of individual neurons to different types of movement here is more complicated than that observed for the vestibular nuclei neurons, perhaps reflective of more complicated cortical processing (78).

### Prolonging Self-Motion Perception: the Velocity Storage Mechanism

**Figure 3:**
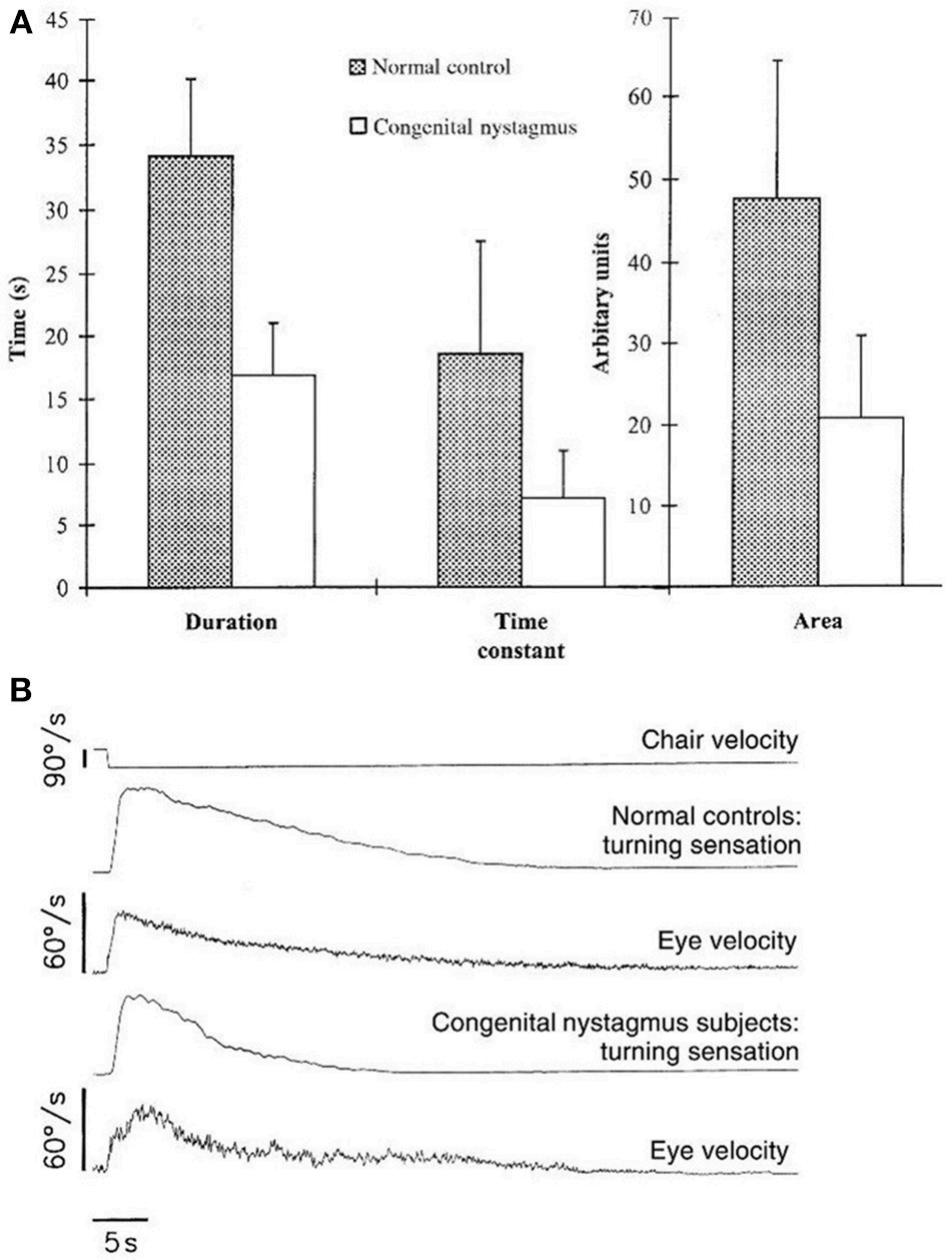
From section Prolonging self-motion perception: the velocity storage mechanism. This figure, from Okada et al. (84). **(A)** Eye velocity and vestibular sensation averaged across subjects (normal, *n* = 31, and congenital nystagmus, n = 14) after suddenly stopping whole-body passive rotations in the dark. **(B)** Mean duration, time constant and area under the curve of turning sensation in subjects with congenital nystagmus and healthy controls.

The use of the relative motion of the endolymph to the bony canals as an indicator of head motion works well for short, fast head movements. However, with prolonged head movements, friction reduces the relative motion of the endolymph, leading to a decay in the signal generated. When the head is rotating at constant velocity, the signal from the semi-circular canals falls to 1/e of its maximum after 3–7 s: i.e., the time constant of the canals lies between 3 and 7 s (79, 80). However, it is conceivable that it might be physiologically disadvantageous for vestibular reflexes and perception to exhibit a similar decay curve, and indeed measured time constants for the VOR and vestibular perception are on the order of three to four times greater than that of the canals (30, 81). The network responsible for prolonging the time constant of the VOR and perception and thus sustaining behavioral responses beyond the time when the endolymph has ceased to move relative to the head is called the velocity storage mechanism. It can be modeled as a leaky integrator with reversal of the sign of the signal and works as a form of imperfect positive feedback on the canal signal to the nuclei (30). The integrator is leaky to prevent inappropriate propagation of noise. The velocity storage network is thought to reside in the cerebellum. Whilst there has been some debate as to whether the VOR and vestibular perception use the same velocity storage network, most work now supports the theory that they share the mechanism. In healthy subjects, there were no differences in perceived rotational velocity and the slow-phase response of the VOR after suddenly stopping yaw and pitch rotations in the dark (82); time constants of the VOR and perceived rotation co-varied in patients with chronic vestibulo-cerebellar degeneration and healthy controls (83); and, when measuring post-rotational nystagmus and perceived rotation using a hand-driven wheel connected to a tachometer, intra-subject group decay time constants for the two variables were the same for healthy controls (16 s), and patients with congenital nystagmus (7 s) (see Figure 3) (84). It is worth noting at this point that the velocity storage network is not the only mechanism responsible for prolonged self-motion perception: the visual system also plays an important role. In general, initial, short-latency responses to self-motion are generated by the vestibular system, whilst responses of greater duration and latency are produced predominantly from visual flow inputs (85).

### Cognitive Cueing

Cognitive, top-down influences are important for many neural processes: self-motion perception is no different. When subjects were asked to imagine themselves rotating in a chair prior to actual rotation, when the imagined and real rotations were in the same direction, vestibular perceptual thresholds were lower and, interestingly, so were thresholds for the VOR (86). Conversely, the ability to generate and manipulate mental images itself relies upon an intact vestibular system: subjects with vestibular impairment performed worse than healthy controls in object-based mental transformations (87). Furthermore, vestibular stimulation can facilitate mental transformations (88), with improved performance during congruent inertial motion (89). Such cognitive cueing is also evident during traditional passive linear self-motion tasks: with sufficient acceleration, one might expect subjects to experience tilt due to the somatogravic illusion. However, this is generally not reported by participants. Wertheim et al found that when subjects have prior knowledge that they will be accelerated from rest during an experiment, they do not report tilt, but that up to 50% of participants report tilt when they have no prior knowledge, suggesting that the sensation of tilt is suppressed in the former group (90).

## So Where Is Self-Motion Perception Processed?

**Figure 4:**
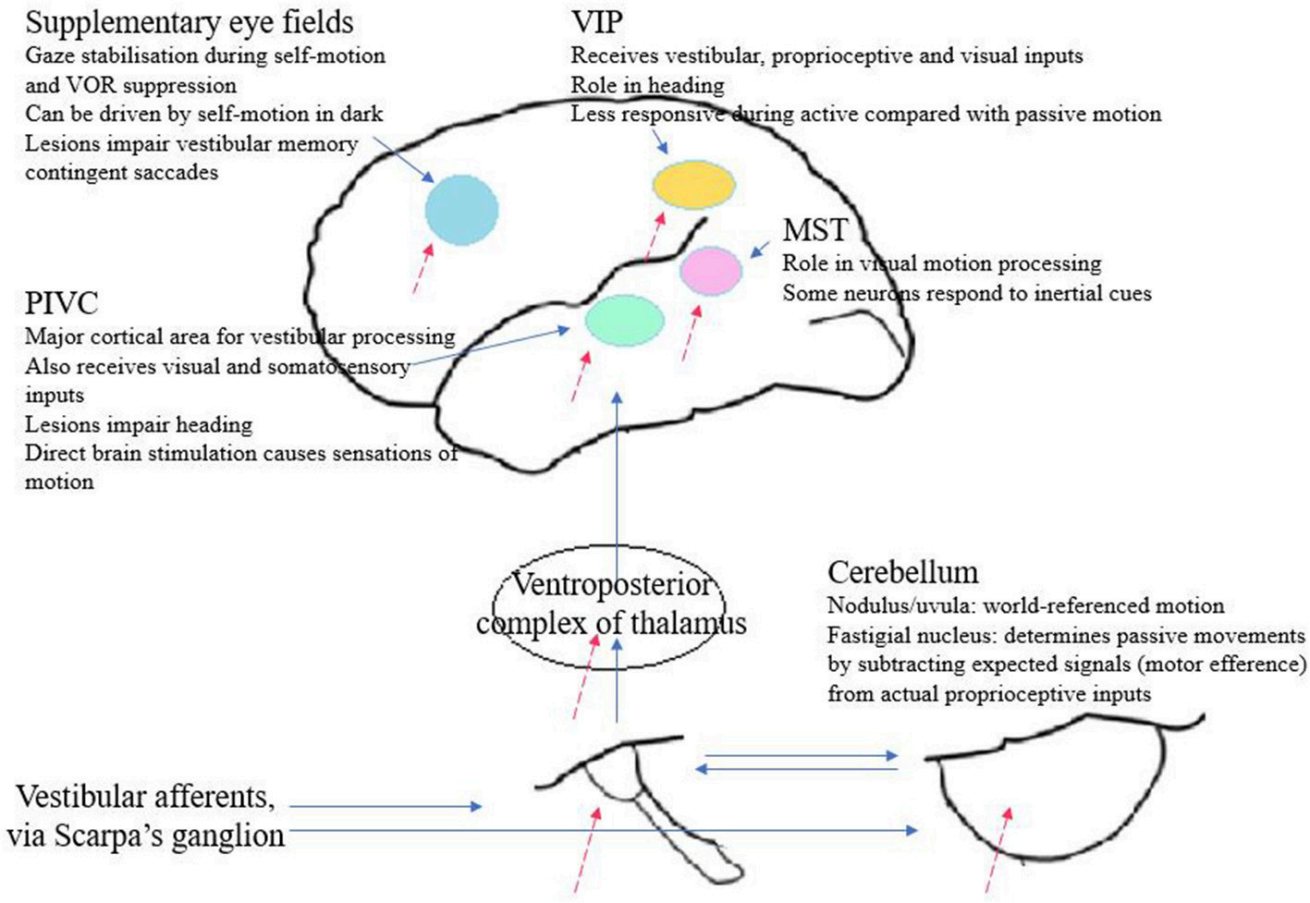
From section So where is self-motion perception processed? Diagram summarizing the main vestibular projections and brain regions contributing to self-motion perception. Dashed arrows indicate integration with extra-vestibular inputs.

Traditionally, perception was thought to be the preserve of the cortex, with sensory inputs passing first through the thalamus and then to a unimodal area of primary cortex before reaching higher association areas to be combined with other sensory inputs. However, this view is changing: more recent findings suggest that multisensory processes occur in primary sensory cortices and recognize the role of non-cortical areas (91). No specific unimodal vestibular cortical area has been identified; rather, cortical neurons that are modulated by vestibular stimuli also respond to visual, proprioceptive, and motor efference inputs. Therefore, the perception of self-motion is believed to be processed by a network of different structures and regions, centered on the lateral fissure and the parieto-insular “vestibular” cortex and including the vestibular nuclei, cerebellum and other cortical areas, a theory that is supported by the multiple areas found to be involved in self-motion perception in animal and human studies. Having a distributed network is of evolutionary benefit as it reduces the risk that a focal brain lesion leads to a significant defect in self-motion perception. Figure 4 summarizes the main components of this vestibular network.

### The Vestibular Neurons and Their Projections

The vestibular fibers, whose cell bodies are found in Scarpa's ganglion, run to the four principal vestibular nuclei in the dorsolateral pons and medulla and directly innervate the posterior cerebellum, as well as projecting to other central structures (92). These nuclei are also interconnected. Many second order vestibular neurons receive convergent inputs from otolith and canal afferents, thus providing a mechanism for early integration of the two signals (93–95). For vestibular-only neurons, so-called as they respond only to change in head attitude and not to eye movement, this appears to occur physiologically in the form of sub-additive integration, with canal afferents more heavily weighted at lower frequencies and otoliths at higher frequencies (72). As an aside, this may be the basis for a correlate seen in human psychophysical experiments, in which perception of combined passive linear and rotational motion cannot be predicted as the simple sum of the two components (96). Vestibular neurons at this level are also modulated by visual and proprioceptive stimuli and from central, top-down inputs (97). The nuclei project to the spinal cord via the lateral vestibulospinal tract and descending medial longitudinal fasciculus; to the autonomic nervous system; to the extra-ocular nuclei via the ascending medial longitudinal fasciculus; to the cerebellum; and, to the thalamus from where there are connections to the cortex (92). A study of patients with acute posterolateral thalamus lesions using positron emission tomography during caloric vestibular stimulation (CVS), demonstrated reduced vestibular temporo-parietal cortex activation on the side ipsilateral to the lesion but did not find any significant effect on motion perception (98).

### Cerebellar Contributions to Self-Motion Perception

Recently, the role of the cerebellum has been recognized as extending beyond the traditional confines of motor control of the eye movements and posture to include sensory discrimination and self-motion perception (99, 100). Anatomically, the nodulus/ uvula and fastigial nucleus of the cerebellum receive significant input from the vestibular system: a smaller contribution is of primary afferent fibers projecting to the ipsilateral uvula and nodulus and a larger proportion of secondary fibers from the vestibular nuclei (101). The vestibular nuclei are reciprocally innervated by the cerebellum. As described above, in monkeys the nodulus/ uvula appear to be important in generation of a world-referenced frame of self-motion (67, 68), whereas the fastigial nucleus generates signals of unexpected self-motion by comparing motor efference signals and actual proprioceptive feedback from movement (76, 77).

**Figure 5:**
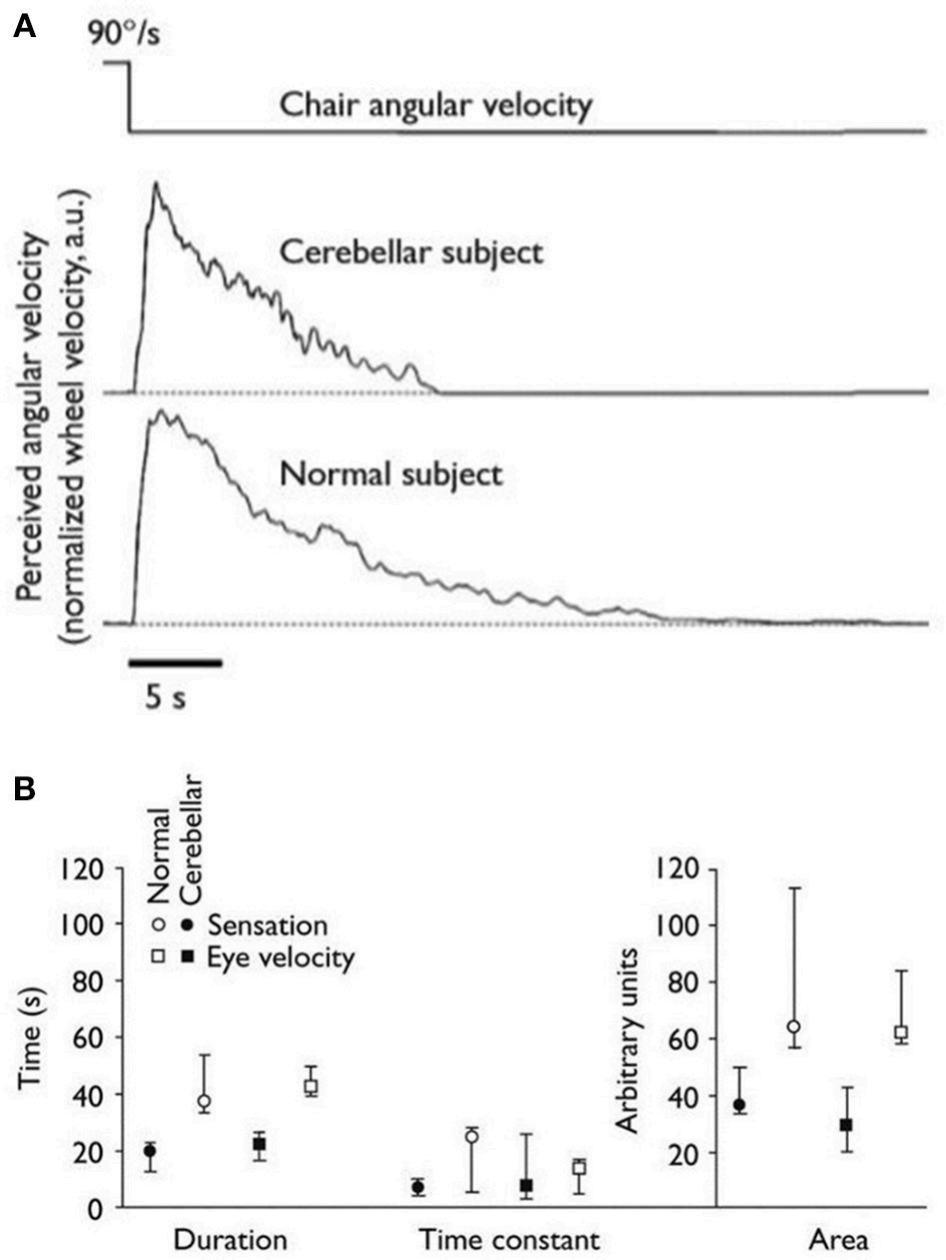
From section Cerebellar contributions to self-motion perception. This Figure, from Bronstein et al. (102). **(A)** Graphs of representative individuals for perceived angular velocity after suddenly stopping rotations in the dark. **(B)** Median duration, time constant and area under the curve for sensation and eye velocity in patients with midline cerebellar degeneration (*n* = 8) and healthy controls (*n* = 8).

In humans, psychophysical studies on subjects with cerebellar degeneration have yielded informative observations: patients with midline cerebellar lesions, when rotated in the dark, showed impairment in multiple parameters compared with healthy controls, including the duration of self-motion perception and the perceptual time-constant (see Figure 5) (102), findings replicated in a subsequent study of patients with chronic degeneration of the vestibulo-cerebellum (83). A further study investigated the vestibular perceptual thresholds of two patients with cerebellar agenesis, finding them to be globally elevated, particularly for movements which only activated the otoliths (103). These observations support the view that the cerebellum has a role in extracting information about self-motion from multiple signals generated by both self and passive movements, and from background noise.

### Cortical Processing of Self-Motion Perception

From the brainstem and cerebellum, vestibular inputs pass through the thalamus, predominantly via the main somatosensory nucleus, the ventroposterior complex, to the cortex. At the thalamus, it has been proposed that information flows in two channels, one encoding head motion, the other body motion (104). Many vestibular-sensitive neurons are already multi-sensory, being modulated by visual, proprioceptive and motor efference signals (105). There are two major cortical areas implicated in the processing of vestibular information for the perception of self-motion: the ventral intraparietal area (VIP) and the parieto-insular vestibular cortex (PIVC). A third, the medial superior temporal area (MST), is critical for visual motion perception but also receives vestibular inputs. The supplementary eye fields appear to be important in the control of eye movements during self-motion: the neurons here are modulated by vestibular stimuli (106); and, patients with lesions of the supplementary eye field exhibited worse accuracy during a vestibular memory-contingent saccade (107, 108).

Neurons in the MST respond predominantly to visual stimuli, and in particular to visual motion stimuli. Several studies have confirmed the importance of this area in visual heading perception (109–112). It has also been shown that a subset of MST neurons are modulated by passive whole-body translations [a response that is not seen following bilateral labyrinthectomy (62)] albeit with smaller and less directionally selective responses than for optic flow stimuli. Combining vestibular stimulation with congruent and incongruent optic flows varied the amplitude and direction-selectivity of these neurons (113). These findings were since extended to show that the response of MST neurons to inertial motion cues was correlated with heading discrimination (62) and that visual and vestibular cues were summed in neurons with congruent heading preferences (114). However, it remains likely that vestibular cues only have a minor influence over the MST area, a theory that is supported by the finding that inactivating MST using muscimol, a GABA_A_ agonist, had little effect on vestibular heading thresholds, but did impair visual thresholds (111). Humans with MST lesions display great difficulty in navigation and have impaired visual motion perception (115).

VIP neurons respond reliably to vestibular, visual and somatosensory inputs (116, 117) and receive inputs from MST (118, 119). Compared with MST, VIP neurons are modulated to a greater extent by vestibular stimuli and show greater correlation with perceptual decisions (120). Subgroups of neurons show preferential response to different types of inertial motion, a characteristic that is invariant with respect to head attitude and gaze direction. It is worth noting that responses to active motion are generally smaller than those to passive motion (116), thus raising the question as to the role the VIP cortex plays in distinguishing active vs. passive motions and coordinating appropriate behavioral responses.

Around two-thirds of PIVC neurons respond to vestibular stimulation (121). Vestibular-responsive neurons are more strongly activated by semi-circular canal inputs compared with those from the otoliths. Of those neurons modulated by canal signals, there were subgroups which preferentially encoded rotations in a specific plane. As noted above, the PIVC is multisensory, and neurons there also respond to somatosensory and (particularly large-field) visual stimuli (122, 123). In primates, lesions of the PIVC led to impaired heading perception (124). In humans, whilst direct stimulation of this cortical area during craniotomies led to a range of vestibular sensations including movement of the world and vertigo (125), cerebral infarctions affecting the PIVC are reported to cause an impairment in subjective vertical (126). Functional magnetic resonance imaging studies have revealed activation of the PIVC and posterior insular cortex during caloric vestibular and direct galvanic stimulation (127–129). During this series of experiments, some conditions required subjects to keep their eyes closed, whilst in others they viewed random movement or a fixation cross. The posterior insular cortex responded strongly to visual motion, whereas in the PIVC there was a trend for visual motion to reduce activity (130). The role of the posterior parietal cortex in self-motion perception is demonstrated in a series of psychophysical experiments. Repetitive transcranial magnetic stimulation to this region impairs performance on a whole-body displacement task that required angular path integration based only on vestibular inputs (131). The same stimulation applied to the right posterior parietal cortex also worsened a motion-reproduction task (i.e., one not requiring path integration) when applied *during* the motion reproduction, although it had no effect when applied during the initial rotation (encoding phase) (132).

The role of the posterior parietal cortex in top-down vestibular perception (as defined by vestibular perceptual thresholds) has also been established: transcranial direct current over the temporoparietal junction (TPJ) alters vestibulo-perceptual and VOR thresholds (133, 134). Nigmatullina et al. found that in ballerinas, who are typically trained to perform multiple pirouettes, display remarkable vestibular adaptation, compared with rowers, who are physically active but not trained to tolerate numerous rotations, there is differential white matter volume in the TPJ bilaterally (135). Lesion studies demonstrate a split in processing dependent upon the nature of the task: subjects with parieto-occipital lesions perform well in “low level” tasks such as discriminating the direction of moving stimuli, but poorly when asked to judge heading direction; the reverse is true for patients with occipital lesions (136). Further studies support this theory of split cortical processing for parallel channels carrying different information about motion (137, 138).

### Lateralisation of Vestibular Cortical Processing

Vestibular stimulation activates both cerebral cortices, but it is recognized that there is a right hemisphere dominance in right-handed individuals, and an even stronger left-sided bias in left-handed subjects (139). Although this asymmetry has been shown to lead to differential effects on both vestibular low-level reflex behaviors including the VOR (140) and on vestibular-sensitive cognitive processes [for example, as recently proposed, anxiety (141)], the effects, if any, on self-motion perception remained to be fully explored. As discussed above, repetitive transcranial magnetic stimulation to the posterior parietal cortex can differentially impair perceived whole-body angular displacement, with worse performance when the right hemisphere is stimulated (affecting leftward rotations) compared with left hemisphere rotations (132). Future work could explore whether control of self-motion perception is more commonly the result of asymmetric or symmetric cortical activity.

## Integration of Vestibular and Non-vestibular Cues in Self-Motion Perception

Whilst the vestibular system clearly plays an important role in self-motion perception, it is far from the only system that can provide such information. This is important because:

- The human body is not rigid and can move with several degrees of freedom, as explored in a novel paradigm establishing relationships in movement between different body parts (142). Thus, the vestibular system alone is insufficient to provide a complete representation of self-motion.
- Using one sensory input alone to perceive self-motion would leave one vulnerable to illusions and false interpretations (for example, the somatogravic illusion in which aviators, deprived of adequate visual stimuli, sense linear forward acceleration as a backwards tilt of the head, potentially leading to the dangerous situation of pitching the nose of their aircraft) and to loss of information (constant velocity is not encoded by vestibular system).
- When using one input, accuracy is worse than can be achieved by integrating multiple inputs.
- Comparison of sensory inputs with motor efference copies enables discrimination between self-generated and passive motion, as described above.

The vestibular system is unusual in that it receives early input from multiple other systems including visual, somatosensory and motor efference signals [reviewed here (97)]. These multisensory inputs enable refinement of self-motion estimates and thereby attune behavioral responses (143). In general, multisensory processing is a skill which improves over time, and self-motion is no different. Supporting this view are recent findings that demonstrate older adults are able to improve their performance in a driving task by a greater margin than younger adults when additional vestibular cues were added to the visual stimulus (144).

### Integration of Visual and Vestibular Inputs

The visual system contributes to self-motion perception, with optic flow-induced perception being highly accurate and precise. Studies in primates reveal that they rely predominantly on the visual system for navigation in three-dimensional space (145). Visual-vestibular interactions occur as early as the vestibular nuclei, although this is mostly seen in neurons involved in the VOR, not in vestibular-only neurons, and thus such interactions are unlikely to be involved in self-motion perception (146, 147). Indeed, Bryan and Angelaki show that VOR neurons in the vestibular and deep cerebellar nuclei cease to respond to optic flow once the OKN was suppressed (by requiring the animals to fixate on a head-fixed target) (147). At higher levels, visual-vestibular input is integrated in cortical areas traditionally associated with visual processing, including the MST and VIP areas. In these regions, there are neurons that respond both to motion in darkness and to optic flow, and the former response is abolished following bilateral labyrinthectomy (62, 148, 149). This may be the neural substrate to explain how combined visual-vestibular stimuli improves self-motion perception compared with either stimulus alone.

When examining the relative contributions of the visual and vestibular systems to self-motion perception, it has become clear that they vary depending upon the experimental conditions rather than having some pre-defined weighting. In a study in which subjects experienced linear acceleration, visual cues enabled more precise determination of heading than vestibular cues (150), whereas in a separate experiment, in subjects undergoing roll rotations, vestibular perception was better at frequencies of sinusoidal motion >2 Hz and visual perception better at frequencies < 1 Hz (151). Kolev et al. rotated supine subjects about the earth-vertical axis, i.e., they underwent roll without otolith stimulation (152). Whilst it is unsurprising that coherent, simultaneous visual-vestibular signals improve perceptual thresholds, the authors found that even conflicting visual-vestibular signals, generated when the subjects fixated on a visual target that rotated with them, yield lower perceptual thresholds than seen with vestibular-alone stimulation (152). The study also demonstrated a frequency-dependence of perceptual thresholds. However, experiments designed to probe the sensation of self-motion as induced by moving visual fields reported visual dominance despite the presence of conflicting vestibular stimulation. That is, in subjects undergoing yaw rotations whilst watching visual fields that were rotating in the same direction but at different velocities, the reported magnitude of self-motion appeared to relate to that of the incongruent visual stimuli (153). In a similar setup, subjects reported self-motion perception in the opposite direction to actual whole-body rotation during prolonged periods of yaw rotation during which the visual field rotating in phase and in the same direction as the vestibular rotation (154). These apparent discrepancies are likely to reflect dynamic reweighting of visual and vestibular cues under different conditions, perhaps reflecting the unlikelihood that information from the visual system is incorrect in daily life.

There is additional cross-talk between the two systems beyond the mere computing self-motion: perceptual learning, as measured by an improvement in vestibular motion discrimination thresholds performed in the dark, occurs when training rotations occur in the light, but not when subjects are blindfolded during training (155).

### Integration of Proprioceptive, Somatosensory, and Vestibular Inputs

Proprioception is another important sensory input used alongside vestibular signals to calculate self-motion. Anatomically, integration of the proprioceptive and vestibular systems occurs directly (dorsal root axons innervate vestibular nuclei) and indirectly (via second order neurons and via the cerebellum) [reviewed in (156)]. Functionally, vestibular-only neurons are modulated by passive neck rotations in squirrel monkeys (157) and in cynomolgus monkeys (158), leading to reduced neuronal activity during head only motion compared with whole-body motion. The latter study found that during passive vestibular and proprioceptive stimulation signals underwent linear summation, but that sub-additive integration occurred during active head movements and during gaze shifts. Such differential processing under different experimental conditions might explain the apparently conflicting finding that during passive movements of the head of the rhesus monkey no modulation of vestibular-only neurons was seen (74). The authors hypothesized that this might be a reflection of the arboreal habitats of squirrel and cynomolgus monkeys compared with the predominantly ground-dwelling rhesus monkey. Proprioceptive-vestibular interactions are also documented in the thalamus, primary somatosensory cortex and ventral intraparietal region (159, 160).

The role of the proprioceptive system in self-motion perception in humans is well-established. During a remembered-target task, subjects performed better when there were combined vestibular and neck proprioceptive inputs compared with the vestibular-alone condition (161). In a similar setup, reducing the stimulus amplitude reduced gain in the vestibular-only condition, but not in proprioceptive conditions (47); the study also found that detection of head turns was predominantly determined by somatosensory inputs (47); and that proprioceptive afferents can reliably encode head on body rotations even when there is no vestibular stimulation (162).

This relationship between the vestibular and somatosensory systems is to some degree reciprocal. Vestibular activation improves sensitivity to tactile stimuli (153, 163–167), possibly via a non-linear mechanism that is only in effect once a certain threshold of self-motion perception has been achieved, and this occurs independently to changes in attention (168). Furthermore, vestibular activation can transiently reverse hemianesthesia secondary to brain lesions, possibly due to altered neuronal dynamics in the putamen, insula and secondary somatosensory cortex (169, 170).

### Optimal Integration

As discussed above, the precision of self-motion perception is greater when more than one sensory input is used. Recently there has been interest into the way in which multi-sensory cues deriving from a common cause are integrated. Across sensory systems, including the vestibular system, data from experiments appear to suggest that inputs are integrated in a Bayesian optimal way, i.e., the weight of each cue is proportional to its reliability (1/variance) (171). Regarding self-motion perception, visual and vestibular cues appear to be optimally integrated during heading discrimination and rotational movements (151, 172–174). Furthermore, the brain can dynamically change the relative weights of cues to reflect changing conditions (173) and can even integrate conflicting sensory cues in a statistically optimal way to minimize variance (172, 175). Recordings from multi-sensory neurons in the dorsal medial superior temporal area point to its role in visuo-vestibular cue integration, with evidence of near-optimal processing (114, 176).

### Aftereffects: Evidence for Shared Hardware to Process Different Stimuli

A method to probe to what degree the same neuronal networks are used to process information from different sensory inputs is to examine cross-modal aftereffects. Aftereffects are the sensations that occur following cessation of the initial stimulus. In the case of motion perception, they typically occur in the opposite direction, thereby shifting perception of subsequent stimuli. For example, in the waterfall illusion, after watching the water drop down toward earth for some time, stationary rocks and trees appear to drift upwards (177, 178). Cross-modal aftereffects refer to sensations that occur in a different modality to the initial stimulus and are thought to represent recalibration rather than a fatigue-induced process as is evidenced by the lack of aftereffects to visual stimuli when they are presented with an appropriate vestibular stimulus (179). When the lights are extinguished following prolonged exposure to a rotating drum, subjects experience self-motion in the opposite direction, an effect which is accompanied by an “after-nystagmus” (180, 181). If the stimulus is not sufficiently long, no aftereffects are experienced: whilst exposure to optic flow inducing a sensation of linear self-motion for 15 s resulted in a shift in perception, shorter durations of up to 7 s had no such effect, even though the onset of vection had occurred by this time (182, 183).

## Vection: An Illusion of Self-Motion

First described in the nineteenth century by Mach (184) and Wood (185), vection is the false perception of self-motion induced by sufficiently large stimuli moving across the retina in the absence of any true acceleration as signaled by the vestibular system. Today, vection is perhaps most recognizably experienced whilst sitting as a rail passenger, looking out of the window and believing that one's own carriage is leaving early, only to realize that it is rather a train on an adjacent platform that is pulling away. The illusion is widely exploited in virtual reality, theme park rides and I-Max cinemas, but it also remains of interest in neuroscience as it reveals details of the relationship of different sensory inputs in the generation of self-motion perception (vection is not solely generated by visual inputs: proprioceptive and auditory stimuli have also been shown to provoke the illusion (186, 187).

Vection is typically experienced several seconds after the onset of the stimulus. For visually-generated vection, there are some general precepts that have been established, with the following all increasing the credibility of the illusion: greater velocity up to a point, previously suggested as 120 degrees/s for a rotating stimulus (188); larger stimulus size (188–190); increased density of moving objects (190); and, circular and curvilinear rather than linear motion (191). Furthermore, Brandt et al. established that it is predominantly the peripheral vision that is responsible for vection: whilst masking the central visual field with black disks, diameters of up to 120° exerted minimal effect on the generation of vection, but when blocking the peripheral vision, central visual stimuli of up to 30° diameter fail to induce self-motion perception; and, when the central and peripheral visual stimuli are of equivalent area, it is the peripheral stimulus that dominates (188). And the perception is remarkably compelling: Brandt et al also demonstrated that subjects still experienced circular vection when the rotating stimulus accelerated at 15°/s^2^ (188).

During vection there is a conflict between incoming visual, somatosensory and vestibular information, with corresponding deactivations in the PIVC during rotational vection (192, 193). In contrast, during linear vection functional magnetic resonance imaging found only activations in various cortical areas with no PIVC or any other cortical deactivations (194). A further study attempted to correlate the intensity and duration of vection with brain activity in different regions. Whilst no correlation was found with PIVC, enhanced activity of the cerebellar vermis and parieto-occipital areas amongst others was reported (195). The authors concluded that this might represent a “dorsal stream” responsible for the intensity of vection. As might be expected given the hypothesized role of the cerebellar nodulus, there is increased activity during periods of reported self-motion illusion compared with object motion (193). Experimentally, subjects with bilateral impaired vestibular function report vection sooner, for longer and more compellingly than healthy controls (196, 197). Such a process of reciprocal inhibition might be explained physiologically as a consequence of a system of flexible dominant sensory weights given to incoming signals which enables self-motion perception during periods of incongruent information (for example, after prolonged rotation when the relative motion of the endolymph has ceased).

As with many perceptions, visual vection can be modulated by the presence or absence of additional inputs. Whilst proprioceptive stimuli alone do not reliably induce vection in all subjects, they can enhance vection. For example when small vibrations are applied to the subjects' seat at the time of onset of visual stimuli (198) or during auditory self-motion illusions (199). Proprioceptive stimuli can also enhance vection induced by auditory stimuli (200) and even static leaning of the upper body can enhance vection (201). In addition, the role of top-down processing and expectation should not be underestimated. It is common practice to “prime” subjects by demonstrating that actual self-motion is possible, even if it will not occur. Work in children demonstrated that linear vection is felt earlier is when a chair is placed on rollers compared with directly on the ground (202).

## How Vestibular Functioning and Self-Motion Perception can Modulate Behavior

Perception of self-motion is critically important for many human behaviors, including heading and navigation and control of body and eye movements. Therefore, it is not surprising that self-motion perception should modulate such behaviors. This section will review the effects of the vestibular system and self-motion perception upon eye movement, postural control and spatial awareness and more abstract behaviors including numerical cognition, human affect and bodily self-consciousness.

### The Relationship Between Self-Motion Perception and Visually-Induced Postural Responses

Lee and Lishmann (203) demonstrated that visual information is important for the control of stance, and visual motion stimuli can induce postural sway (visually-evoked postural response, VEPR). The VEPR is known to be influenced by stimulus size and displacement across the retina (204) and it would appear logical that information containing cues regarding self- vs. object-motion would also modulate sway. Using transient movements of a visual scene to induce a postural response, Guerraz et al. (205) showed that sway was reduced when the subject could control some aspect of the stimulus motion compared with the uncontrolled condition. Moreover, in an oscillating room paradigm, when participants are aware that there is object-motion rather than self-motion, not only do they sway less than subjects who are unaware, they also do not show any change in sway as this distance between them and the wall increases (206). The authors also observe that the variability within each subject group was the same, and concluded that the prior information leads to a reweighting of different sensory cues in the control of posture. In further experiments, (static) subjects viewed a horizontally-translating background with either a head-mounted or earth-fixed LED at the center of a luminescent window frame (207). In these scenarios, the direction of postural sway depends upon the nature of the foreground, being in the direction of the background motion for the head-fixed display and transiently reversed in the earth-fixed case, whilst vection only occurred in one direction (opposite to that of the background motion). Subjects experienced vection sooner and for longer in the head-fixed condition. As vection is delayed compared with the VEPR, and as it is unidirectional compared with the bidirectional VEPR, it is likely that the two are processed differently. However, when subjects were experiencing self-motion there was significantly greater sway in both conditions as measured by displacement at C7 level, an effect that preceded vection onset (as indicated by pushing a button) by ~1 s. The authors argued that there may be a dual system at work, similar to that governing the eyes and reviewed here (85), in which as short-latency, brief VEPR (responsive to parallax) is subsequently replaced by a longer-latency visuo-postural response that can be enhanced by vection and might control posture during prolonged body displacements.

### Self-Motion and the Detection of Movement

Whilst freely walking, one perceives the world to be stationary despite its projection moving across one's retina, and additionally, moving objects are perceived as moving, the result of subtracting expected inputs from actual inuts, discussed above and reviewed by DeAmgelis and Angelaki (208). The thresholds at which object motion can be detected, as well as the reaction times for such visual perception, are, however, increased during self-motion as compared with when the subject is stationary (209). Conversely, the threshold for vestibular perception are increased when subjects simultaneously view a moving visual pattern (189). Furthermore, when viewing a bistable rotating Necker cube, participants perceived the cube to be rotating in a congruent direction with their own passive whole-body rotation (210), and when viewing a bistable plaid, in which the observer perceives either two gratings moving across each other, or a single percept moving coherently, self-motion modified the dominance of each percept such that when self-motion and the global coherent percept were in opposite directions, the dominant percept was of a coherent image, and when self- and global percept- motion were orthogonal, subjects were more likely to view the image as two gratings moving independently (congruent motion had no effect) (211). The authors of the latter study suggest that this occurs as a result of an interaction between the visual motion and self-motion vectors at the stage of motion integration.

### Self-Motion Perception and Spatial Awareness

Spatial representation within the brain has been the focus of much research over the last 70 years, and the vestibular system plays an important role in tracking and updating one's location in space reviewed by Moser et al. (212, 213) and Fyhn et al. (214). It might be noted here that this role is not limited to space as defined by visual inputs: the construct of auditory space is also dependent upon self-motion and it was recognized in 1940 that, despite movement of the head, human subjects can perceive a stable auditory environment and use it to accurately localize sounds (215). More recently, experiments have demonstrated that auditory space can be distorted by passive and active self-motion, with constructed space shrinking during forward acceleration (i.e., subjects indicate that sounds are located as being physically further away from them during periods of forwards acceleration compared with the stationary scenario, a phenomenon that has a dose-dependent relationship) (216, 217). For the purposes of this review, we will focus on the role of the vestibular system in: the perception of vertical, the modulation of visuospatial attention, with particular reference to patients experiencing visuospatial neglect, and visuospatial memory and navigation.

#### The Perception of Vertical and Vestibular Dysfunction

Verticality can be perceived through via visual, somatosensory and vestibular cues, and it follows that such perception can be affected by vestibular dysfunction. Following peripheral vestibular lesions, humans tilt their head, and shift their center of mass toward the side of the lesion (218, 219). Vestibular lesions have dissociative upon the perception of verticality dependent upon the experimental paradigm: whilst the subjective visual vertical was strongly deviated toward the side of the lesion in patients, the subjective seated postural vertical was not significantly different between the patient and control groups (220) [perception of the static visual vertical typically returns around 1 year after the insult (221, 222)]. The deviation in visual vertical is likely explained by altered inputs from the otoliths, leading to an altered representation of the gravitational vector and disturbance of the subjective visual vertical (223), and indeed, patients with bilateral peripheral vestibular dysfunction have been observed to have normal subjective visual vertical (224). This latter patient group also have a preserved postural vertical, although the sensitivity of this is reduced in patients who have a fluctuating (as opposed to stable) abnormality in vestibular dysfunction (225), suggesting that proprioceptive and somatosensory inputs are important in this perception, with the vestibular system refining the estimate for verticality. Subjective visual vertical can be improved, although not normalized, by the presence of visual cues for horizontal and vertical, for example as are found in an ordinary room (226). In the same experiment, Borel et al. found that the postural tilt toward the size of the lesion was reversed in the condition when visual cues were provided. Estimates for visual vertical also improve when subjects are balancing in a precarious position, for example on a beam, leading to the hypothesis of the “dynamics of balance,” that is, that we have a heightened awareness of our orientation the more unbalanced we are (227). These findings are reviewed by Lopez et al. (228), who propose that the changes seen in relation to patients' perception of verticality following peripheral vestibular lesions are adaptive and might be explained in terms of changing the frame of reference (gravitationally-, egocentrically- or allocentrically-orientated) and of higher postural constraints. The neural substrate underpinning such reference frames is suggested to be a distributed neural network including the premotor cortex, premotor cortex, inferior parietal lobule, posterior parietal cortex, insula, and the temporo-parietal junction (228).

#### The Effect of Self-Motion Perception on Gaze Direction and Optokinetic Nystagmus

During self-motion perception, as compared with visual field motion without self-motion perception, there is a shift of the mean gaze direction toward the incoming visual stimulus, which reverts when the perceptual state reverts to object motion (229, 230). The change in mean gaze direction may be viewed as a shift in visuospatial attention during times of perceived self-motion (230). It is worth noting that this shift in gaze toward the incoming visual stimulus is seen when subjects are instructed to passively stare at the rotating stripes [in contrast, subjects actively pursuing the visual stimulus undergo a shift of mean gaze in the direction the stimulus is moving toward (231)] and thus the subtleties of human behavior modulation, reflecting underlying perceptual strategies, are revealed.

Thilo et al. (230) also found that shifts in perceptual state were also linked to changes in the slow-phase gain of optokinetic nystagmus (OKN), with self-motion associated with reduced gain, possibly as a result of conflict between the need to accurately pursue the visual stimulus (moving in one direction) and the drive of the eye toward the incoming visual field (in the other direction). Additionally, slow-phase gains were generally decreased when the subjects were supine compared with upright (all subjects viewing the same stimulus rotating about their longitudinal axis). This is the opposite finding to earlier work that showed enhanced slow-phase gains in the supine position compared with the upright position when the optokinetic stimulus was rotating about the subjects' naso-occipital axis, generating a torsional OKN (232). The authors postulate that this differential response is a result of the presence or absence of conflict between information arising from the otoliths and the visual system: in times of conflict, the otoliths may exert an inhibitory influence on the OKN.

#### Visuospatial Neglect and the Vestibular System

Neglect is the clinical phenomenon whereby patients fail to respond to, report or orient toward stimuli on the contralesional side (233). It can be multimodal and includes visuospatial, auditory, and somatosensory neglect. In one study, Bisiach and Luzzatti found the neglect can even affect internal visualization: in patients asked to recall the Piazza del Duomo in Milan, when imagining the scene with their backs to the cathedral, they were observed to omit places on the left side of the scene, places that they subsequently named without prompt when asked to imagine the same scene from the other side of the piazza, facing the cathedral (234). Neuroanatomically, neglect is particularly associated with lesions of the right posterior parietal cortex, including the TPJ (235). Perhaps it is not so surprising then that stimulation of the vestibular system, which is intricately linked with the TPJ, can modulate neglect. First reported in 1941, left-cold and right-warm CVS temporarily alleviates left visuospatial neglect, an effect which appears to be related to shift of spatial attention to the left and facilitation of left lateral gaze (236–238), and functional MRI during left-cold stimulation does demonstrate activation of the right hemisphere (239). Galvanic vestibular stimulation appears to have a similar effect, with right-cathodal stimulation most effectively improving line bisection error in patients with neglect (240). These findings have been extended by work demonstrating that optokinetic stimuli can also improve performance in behavioral tests of neglect (241–243), improvements that have been reported to last for up to 2–4 weeks after treatment (244, 245). However, whilst performance on behavioral tasks might improve following optokinetic stimulation in patients with neglect, there is evidence to suggest that such stimulation does not correct the suspected underlying asymmetry of spatial representation in the brain. Leftwards optokinetic stimulation improved performance in line bisection, but accentuated the leftward bias that patients had when asked to construct a line of known length on the basis of a given “midpoint” (246).

#### Vestibular Dysfunction, Visuospatial Memory and Navigation

That the vestibular system might play a role in spatial memory is suggested by neuro-anatomical studies which demonstrate connections between various vestibular centers and the hippocampus, where so-called place cells are found, (9, 247) and supported by functional MR imaging during CVS (248). And whilst it is evident that the vestibular system is responsible for simple navigation tasks in the absence of other cues (for example, estimation of passive rotational and linear displacements in silence and in dark), it has only been more recently demonstrated that such impairments extend to more complex navigational tasks. Peruch et al. allowed subjects to explore a path using either proprioceptive-vestibular, visual-vestibular, or visual-alone inputs and then asked them to reproduce or reverse it or to take a “shortcut” back to the start in the same environment (249). Patients with unilateral vestibular impairment did much worse in the visual-alone and visual-vestibular conditions, the deficit being more marked the more complex the task. The fact that performance is impaired even in the visual-alone paradigm is perhaps surprising: patients with vestibular impairment might have been expected to perform better than their healthy peers in view of upregulated visual pathways to compensate for their vestibular loss. Yet the findings have been replicated in an experiment using a virtual Morris water task to test spatial memory and navigation, and furthermore that patients with bilateral vestibular loss have significant specific hippocampal atrophy compared with healthy controls (9) and also in animals (250, 251), lending further evidence that the vestibular system is important in spatial memory and navigation. Adding to this evidence is the observation that left-cold CVS significantly improved performance in an object-location-recall task (252).

### The Vestibular System and a Sense of Self

Bodily self-consciousness, which comprises of self-location, self-identification and first-person perspective, is one of the higher-order functions influenced by the vestibular system. Bodily self-consciousness is thought to be the summation of different sensory and motor efference inputs, including that of the vestibular system, that allows for the construct of personal space (i.e., the space occupied by the body and the space immediately surrounding the body) and of extrapersonal space, reviewed by Blanke (253). Evidence for the role of the vestibular system in the construct of bodily self-consciousness also comes from patients with vestibular impairment: it is well-recognized that patients with vestibular dysfunction can experience a range of abnormal sensations, from distorted body image and schema to depersonalization, derealisation and out-of-body experiences, observations that were first recorded a century ago by Bonnier (254) [republished (255)] and Schilder (256). The role that the vestibular system plays in each of these symptoms is reviewed by Lopez and by Pfeiffer and for a fuller account the reader is directed to (257, 258).

Altered bodily self-consciousness has been linked with changed perception of body parts: patients with vestibular impairment reported changes in how various body parts feel during episodes of dizziness (254, 256) and caloric and galvanic vestibular stimulation has been observed to modify healthy subjects' perception of hand size (259, 260). Additionally, body integrity image disorder, which describes a syndrome in which patients complain of a mismatch between how they feel and how they physically are, with the result that they often request limb amputation, and somatoparaphrenia, in which patients, following a right parietal stroke, reject their left arm as being alien, can be improved temporarily by CVS (261, 262).

Depersonalization, the sensation of being detached from oneself, and derealisation, that of being detached from one's surroundings, are also thought to be a consequence of disturbed bodily self-consciousness. Not only have these symptoms been documented in patients with vestibular dysfunction, but Jáuregi-Renaud et al. found that the depersonalization/derealisation scores of patients as measured by Cox and Swinson's questionnaire were correlated with their error in estimation of passive whole-body rotation (263, 264). Symptoms of depersonalization and derealisation can also be induced in healthy controls by CVS (265). Although the neural correlate of such symptoms has yet to be conclusively identified, the superior temporal gyrus and TPJ seem to be the strongest candidates: electrical stimulation of this area caused subjects to report that they felt strange [for review, see (266)]; patients with depersonalization/derealisation symptoms had altered metabolism here on positron emission tomography (267); and, repetitive transcranial magnetic stimulation of the right TPJ has been reported to alleviate these symptoms (268).

Out-of-body experiences are also associated with disturbed bodily self-consciousness. Such experiences typically have three characteristics: the person feels that they are in an illusory body that is removed from their physical body and that they have a first-person perspective of looking back at their physical body. They have been linked with a TPJ dysfunction, most often affecting the right side (269, 270). Sufficiently strong electrical stimulation of that region of the cortex also induces the illusion, with lower levels of stimulation inducing a sensation of falling or sinking (271). Out-of-body experiences have been observed to occur most typically when subjects are in a non-upright position (272), which is proposed to be due to visual-vestibular conflict, the otoliths, by signaling the direction of gravity, normally being important for forming a strong world-referenced image of oneself (273). Out-of-body experiences can also be induced by combined visual-vestibular-somatosensory conflict, for example, in healthy subjects watching the back of a virtual body being stroked whilst feeling a synchronous stroking on their own back (274). In such cases, interfering with TPJ functioning using transcranial magnetic stimulation abolished the illusion, yet the ability to imagine transformations of external objects was unaffected, suggesting that the TPJ performs a specific role in the processing of self in space and of bodily self-consciousness (275).

Interestingly, patients with schizophrenia have been observed to have a degree of vestibular dysfunction and reduced functional connectivity of the vestibular system (276–278). Schizophrenia can be thought of as a disease of impaired multisensory processing with symptoms of depersonalization, derealisation, distorted first person perception, and loss of agency. The onset of psychosis is often preceded by a period of social withdrawal and sub-delusional detachment from reality. The psychotic period of the illness was defined historically by the presence of Schneider's first rank symptoms of delusional perception, auditory hallucinations, and delusions of thought interference and passivity. The TPJ has been implicated in auditory hallucinations (279, 280), with reported symptom improvement following repetitive transcranial magnetic stimulation (281) and reduced TPJ-hippocampal connectivity has been associated with poorer social performance and negative symptoms (282, 283), reviewed by Wible (284). CVS has been recorded as improving insight into illness in schizophrenia (285) and reducing delusions in schizoaffective disorder (286).

### The Vestibular System and Human Affect

Patients with vestibular impairment have been observed to suffer from a high burden of psychiatric disease, particularly affective disorders such as anxiety and depression (287–290). Moreover, patients with psychiatric disease and no diagnosis of vestibular impairment have been found to have abnormal behavioral responses in tests known to rely upon intact vestibular functioning, including postural control (291) and vestibulo-oculo-motor tasks (292), and even in healthy subjects, mood state modifies balance control (293). It is thought, therefore, that the increased burden of psychiatric disease amongst patients with vestibular impairment compared with the general population might be explained by more than just the observation that chronic disease can negatively impact upon mood. Neuro-anatomically, there are cortical areas that are known to process vestibular information and to be involved in the regulation of mood and affect, including the anterior cingulate cortex (ACC) (294, 295). CVS has been shown to modify activity in the ACC and left-cold stimulation has been shown to increase risk estimation (and reduce unrealistic optimism) (296), improve anosognosia (a syndrome in which patients with evident disability deny any illness) (296) and modulate affective control and mood (297). Furthermore, positron emission tomography has revealed increased activity in the ACC of patients with mania associated with bipolar disorder and euthymic controls (298), and CVS has been reported to temporarily reduce the symptoms of mania in such patients (286, 299). The role of the ACC and vestibular stimulation in depression has yet to be fully investigated, although one might note that there are reports of abnormal eye movement control in depressed subjects (300). Chronic pain, itself associated with changes in mood and affect, is thought to be partly mediated by C-nociceptor input to the ACC, and in some patients, CVS has been reported to reduce symptoms (301, 302).

### Self-Motion Perception and Recovery From Vestibular Dysfunction

After an insult to the vestibular system, it is usual for there to be abnormalities of low-level vestibular functions, such as the VOR, as well as of higher-order functions, such as motion perception. In such patients, and after a certain delay, it has been observed that whilst vestibular perception may have returned to normal or near-normal, the lower-level functions may remain abnormal, so-called perceptuo-reflex uncoupling, suggesting that, under optimal circumstances, higher-order processing can compensate for vestibular dysfunction (48, 135). Such compensation need not be the preserve of recovery from illness: as mentioned above, ballerinas who are accustomed to performing multiple pirouettes demonstrate similar perceptuo-reflex uncoupling (135), an uncoupling that has been proposed to occur via cerebellar sensory gating (77, 135, 303). [The reverse, symptoms of dizziness without abnormal VOR is, of course, a well-known phenomenon seen in brainstem infarction, epileptic seizures and electrical stimulation (126, 304). It is possible that in patients who go on to develop chronic symptoms after an initial vestibular insult, there is an impairment in such central compensatory processing. Such patients typically demonstrate poor correlation between VOR function (which is often unremarkable on clinical testing) and their symptoms (52). Research suggests that factors for good recovery from vestibular lesions include anxiety, visual dependence, autonomic arousal, depression, and fear of bodily sensations (54), and whilst some of these might be viewed as contributing to a psychological component to their symptoms, as summarized above, the co-existence of vestibular disorders and anxiety may point to shared central pathways. A better understanding of these might improve identification of such patients and clinical management of their disease.

### The Effect of Self-Motion Perception on Numerical Magnitude Allocation

**Figure 6:**
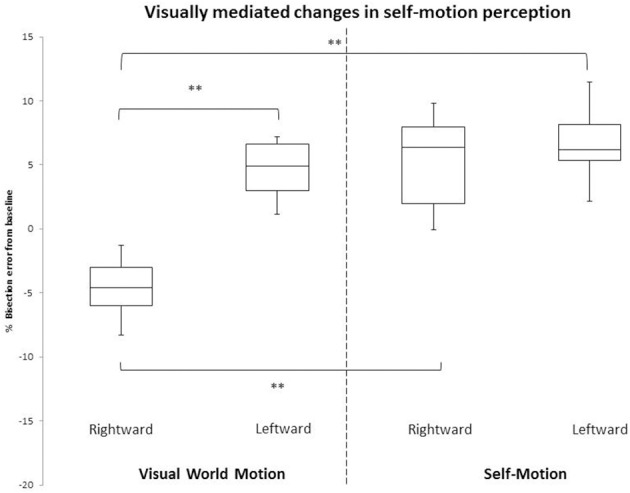
From section, The effect of self-motion perception on numerical magnitude allocation. This Figure, modified from Arshad et al. (306). Graph showing percentage error in the number bisection task (normalized to 0% error by subtracting the baseline) for the four perceptual conditions: world motion right and left and self-motion (vection) right and left. Box-plots represent the median and interquartile range with whiskers denoting 10th and 90th percentile. ^**^Marks significance at *p* < 0.01.

Other more abstract influences of self-motion on behavior include the relationship between numerical magnitude allocation and perception of self-motion. This is interesting not least because, at first consideration, the two processes might appear to be relatively independent. It is worthwhile acknowledging here that the exact relationship between numerical representation in the brain and visuospatial attention is debated (305, 306). Nevertheless, various experiments investigating the effect of self-motion on magnitude allocation have been carried out. Evidence supporting the hypothesis that numerical magnitude allocation can be influenced by self-motion includes: the bidirectional relationship between numerical magnitude and self-motion perception thresholds observed in subjects undergoing whole-body linear motion (307); modulation of the spontaneous number generator by lateral head turns and galvanic vestibular stimulation (308, 309); and, in stroke patients with visuospatial attentional biases (who have been shown to have concomitant biases in numerical estimations (310) viewing a visual stimulus moving toward the side of the neglect temporarily reversed the numerical bias (311, 312). In a recent study examining the effect of perceptual state of self during motion on a mental number-pair bisection task (estimating the mid-point between two numerical values), it was found that: vestibular-alone stimulation exerted no differences in number-pair bisection compared with baseline; when the subject perceives the world to be moving and themselves stationary, rightwards motion reduced the magnitude of estimates compared with baseline and leftwards motion increased the magnitude; and, during vection, both leftwards and rightwards vection elicited the same increase in magnitude of estimates as leftwards world motion, a finding explained by the inhibition seen in the right vestibular cortex during vection and thus leading to left hemisphere dominance and biasing toward larger numbers (see Figure 6) (306).

### Self-Motion Perception and Economic Decision Making

Related to the role of the vestibular system in numerical magnitude allocation are the recent findings that implicate vestibular stimulation in economic decision making (297). Purchase decision making describes the motives and considerations involved in buying a product and include the desirability of that product as well as its cost and the maximum the individual is willing to pay (313–315). In their experiment, Preuss et al. had subjects choose to buy or not to buy products (listed at 20% of the market price) either during sham or left-cold CVS. In the second half of the experiment, subjects ranked the desirability of products and their own “willingness to pay” for those products when the products were displayed at a range of prices, up to 100% of the market price. During left-cold CVS, subjects were less likely to buy products, and they also rated products as being less desirable. In contrast, the willingness of the subjects to pay for those products was not significantly different during sham and stimulation conditions.

Also probing the effects of the vestibular system on economic and prosocial decision making, Arshad et al. (316) used a modified version of the dictator game and a non-numerical prosocial questionnaire probed the effects of vestibular stimulation and binocular rivalry on participants' strategies. They found that there exists a correlation between inherent number-pair bisection error and the mean amount of money a subject donated to an unknown stranger, and that modulating numerical magnitude perception through combined CVS and binocular rivalry led to congruent changes in the mean amount donated, and that this occurred in a proportional manner. The intervention had no effect on the results of the altruism questionnaire, suggesting that the effect was mediated via numerical magnitude. The neural mechanism for such behavior remains to be determined, although a role for the ACC has been hypothesized (316).

## Conclusion

The vestibular system may have developed as an organ to sense movement and coordinate postural and eye reflexes designed to stabilize the body, but its role in the generation of perception of self-motion, though less well-recognized, is equally important. Our understanding of the neurophysiology of self-motion perception has increased over the past few decades through a multitude of electrophysiological studies, psychophysical experiments, and observations from clinical medicine. Vestibular afferents undergo early processing and integration with somatosensory, visual and motor efference inputs in the vestibular nuclei. Such processing is evident throughout the vestibular network including in the cerebellum where it contributes to generation of world-framed motion and unexpected motion and in the PIVC and VIP where heading perception is processed. Future work might focus on the effects of different perceptual states on higher cognitive processes, perhaps examining the role, if any, that vestibular cortical lateralisation plays, and in doing so discover better tests for the monitoring of patients with central and peripheral vestibular disorders which may open up new avenues for the treatment of these diseases.

## Author Contributions

ZB conceptualized and wrote the manuscript. QA conceptualized and edited the manuscript.

### Conflict of Interest Statement

The authors declare that the research was conducted in the absence of any commercial or financial relationships that could be construed as a potential conflict of interest.
